# Regulatory role of circular RNAs in the development of therapeutic resistance in the glioma: A double-edged sword

**DOI:** 10.22038/ijbms.2024.81644.17669

**Published:** 2025

**Authors:** Negin Masoomabadi, Ali Gorji, Tahereh Ghadiri, Safieh Ebrahimi

**Affiliations:** 1 Department of Neuroscience and Cognition, Faculty of Advanced Medical Sciences, Tabriz University of Medical Sciences, Tabriz, Iran; 2 Shefa Neuroscience Research Center, Khatam Alanbia Hospital, Tehran, Iran; 3 Neuroscience Research Center, Mashhad University of Medical Sciences, Mashhad, Iran; 4 Epilepsy Research Center, Münster University, Münster, Germany; 5 Department of Clinical Biochemistry, Faculty of Medicine, Mashhad University of Medical Sciences, Mashhad, Iran

**Keywords:** Cancer therapy, Chemoresistance, CircRNAs, Drug resistance, Glioblastoma, Glioma, Noncoding RNA

## Abstract

Gliomas are the most common lethal tumors of the brain associated with a poor prognosis and increased resistance to chemo-radiotherapy. Circular RNAs (circRNAs), newly identified noncoding RNAs, have appeared as critical regulators of therapeutic resistance among multiple cancers and gliomas. Since circRNAs are aberrantly expressed in glioma and may act as promoters or inhibitors of therapeutic resistance, we categorized alterations of these specific RNAs expression in therapy resistant-glioma in three different classes, including chemoresistance, radioresistance, and glioma stem cell (GSC)-regulation. circRNAs act as competing endogenous RNA, sponging target microRNA and consequently affecting the expression of genes related to glioma tumorigenesis and resistance. By doing so, circRNAs can modulate the critical cellular pathways and processes regulating glioma resistance, including DNA repair pathways, GSC, epithelial-mesenchymal transition, apoptosis, and autophagy. Considering the poor survival and increased resistance to currently approved treatments for glioma, it is crucial to increase the knowledge of the resistance regulatory effects of circRNAs and their underlying molecular mechanisms. Herein, we conducted a comprehensive search and discussed the existing knowledge regarding the important role eof circRNAs in the emergence of resistance to therapeutic interventions in glioma. This knowledge may serve as a basis for enhancing the effectiveness of glioma therapeutic strategies.

## Introduction

Gliomas are the brain’s most common lethal tumors, representing 81% of the central nervous system (CNS) malignancies that typically arise from astrocytes, ependymal cells, and oligodendrocytes (1-3). The WHO grades for gliomas include low-grade or benign tumors (grade 1 and grade 2), which are considered to be low proliferative tumors, and high-grade tumors (grade 3 and grade 4), which are regarded as high proliferative tumors (3, 4). Among brain tumors, glioblastoma (GBM) as a grade IV glioma is the most lethal tumor of the CNS (4). The median survival time in patients with low-grade glioma is about 11.6 years, whereas in patients with grade 3 glioma is about three years, and grade 4 glioma patients have a low median survival time of 14 months(5, 6). None of the applicable therapeutic options for gliomas, including surgery, chemotherapy, radiotherapy, and a combination of them, has been shown to treat glioma completely so far (7). 

Despite recent substantial work to develop efficient therapeutic approaches, the poor prognosis of glioma has not been improved, and the emergence of resistance to chemo-radiotherapy remains a serious issue (8). Therefore, there is a need for further identification of the molecular processes implicated in glioma oncogenesis and therapeutic resistance. Gene mutations (9, 10), activation of cell survival and DNA repair pathways (11, 12), upregulation of ATP-binding cassette (ABC) transporter proteins (13), the existence of glioma stem cells (GSCs) (14), the stimulation of autophagy (15), epithelial-mesenchymal transition (EMT) (16), and epigenetic regulation have been proposed as potential underlying mechanism of the resistance (17). As an important part of epigenetic regulation, noncoding RNAs (ncRNAs) are crucial for modulating therapeutic resistance (18-20). A specific and newly identified ncRNA molecule is circular RNA (circRNA). CircRNAs are single-stranded RNA constituting a circle via covalent binding of their 5′ and 3 ends (21, 22). CircRNAs have a long half-life, high conservation, and distinct expression patterns according to cell-tissue type and developmental stage (23, 24). Growing evidence supports the abnormal expression of circRNAs in cancers, including glioma, which may operate as a double-edged sword in therapeutic resistance, enhancing and inhibiting resistance (25, 26). Indeed, they play a dual role as either oncogenic circRNAs (onco-circRNAs) or tumor suppressors (27). Tumor suppressor circRNAs are down-regulated in the tumor cells, while onco-circRNAs are highly expressed and enhance therapeutic resistance by regulating EMT, CSCs, apoptosis, and autophagy (28-30). Mechanistically, circRNAs can either act as “sponges” for microRNAs (miRNAs) as major regulators of post-transcriptional genes or act as competitive endogenous RNAs (ceRNAs) to modulate parental gene expression related to the tumorigenesis and resistance (24, 31). Considering the poor survival and enhanced resistance to current treatments for glioma, a deeper understanding of the resistance regulatory effects of circRNAs and their underlying molecular mechanisms is necessary to improve the efficacy of current therapeutic methods. Therefore, in this paper, we particularly discussed the existing knowledge concerning the regulatory role of circRNAs in developing resistance to therapeutic interventions in glioma. 

**Overview of CircRNAs characteristics and implication in glioma pathogenesis**

**Figure 1:**
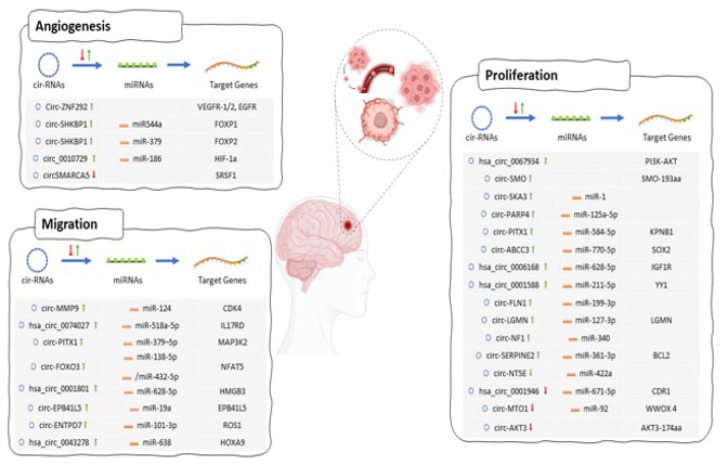
A diagrammatic illustration of circRNA-miRNA-target gene interactions involved in glioma tumorigenesis

Recent developments in high-throughput sequencing approaches and computational methods have revealed ncRNAs in many cell types and tissues. ncRNAs, including long noncoding (lnc)RNAs, miRNAs, and circRNAs, play important roles in tumorigenesis and resistance. CircRNAs are newly identified ncRNAs produced in the circular form by back-splicing premature messenger RNAs (mRNAs) (21-23). Various kinds of circRNA, such as exonic or mixed exonic-intronic, can be produced due to RNA back splicing. Since circRNAs have a closed circular structure, they are stronger than linear RNAs and resist exonuclease-mediated degradation (24). Furthermore, they are abundant in the human transcriptome, highly conserved, and typically have cell type and tissue-specific expression patterns (32). CircRNAs act as miRNA or protein sponges and regulate mRNA transcription and protein-coding (24). Moreover, they can participate in intercellular communication by entering exosomes (33). These specific characteristics of circRNAs reveal their promising diagnostic, prognostic, and therapeutic potential. Accordingly, differential expression analyses have identified the differentially expressed circRNAs in glioma cells and tissue specimens compared with the parallel normal tissues (34, 35). Aberrant expression of circRNAs plays critical roles in tumor cell proliferation, angiogenesis, invasion, and metastasis by regulating the complementary miRNAs and/or target mRNAs of different oncogenic signaling pathways, consisting of nuclear factor-kB(NF- kB), Notch, Janus kinase/signal transducer and activator of transcription (JAK-STAT), Wnt (wingless)/β-catenin, tumor growth factor β (TGF-β), and phosphoinositide 3-kinase (PI3K)–AKT pathways (36-39). The role of circRNAs in glioma progression and pathogenesis is vast and beyond the scope of this review. Interested readers are referred to references for a detailed review of this topic (40-42). This review focuses on circRNA-miRNA-target gene interplays engaged in chemo/radioresistance; however, we summarized some circRNA-miRNA-target gene interplays engaged in glioma tumorigenesis in Figure 1. The following sections will thoroughly discuss the regulatory effects of circRNAs in resistance to chemo/radiotherapy.

**CircRNAs involved in glioma chemoresistance (Temozolomide)**

Temozolomide (TMZ) is an oral bioavailable DNA-alkylating anticancer drug frequently exploited as the standard first-line treatment for GBM by initiating cellular DNA damage in glioma cells. However, most glioma patients develop resistance to TMZ due to increased DNA repair and dysregulation of apoptotic-related genes and signaling pathways (43). Recent advancements in oncology have identified additional novel resistance mechanisms, including the activity of drug efflux transporters, the role of miRNAs, activation of cellular autophagy and associated cell survival, and the emergence of stem cells. Microarray experiments suggest the different profiles of circRNAs in TMZ-resistant glioma and the corresponding primary tissues. High expression of specific circRNAs is deeply intertwined with the resistance of TMZ (44). Mechanistically, all these circRNAs can exert their functions by competitively attaching to miRNAs and further modulating the expression of genes involved in glioma tumorigenesis and resistance. Consistently, hsa_circ_0000936 is up-regulated in TMZ-resistant glioma cells, and hsa_circ_0000936 operates as a miR-1294 sponge affecting TMZ sensitivity (45). Wei *et al*. explored the potential function of circASAP1 in modulating the TMZ resistance of GBM (46). They observed that circASAP1 expression was highly increased in TMZ-resistant cells and recurrent GBM tissues.CircASAP1 up-regulation increases TMZ resistance via induction of cell growth and inhibition of cellular apoptosis so that knockdown of circASAP1 could reverse these effects and ultimately enhance the TMZ sensitivity in both *in vitro* and* in vivo* experiments. Furthermore, data suggests that eukaryotic translation initiation factor 4A3 (EIF4A3) binds to a circASAP1 flanking sequence and further increases circASAP1 expression (46). The role of circASAP1, as a competitive sponge for miR-502-5p, is implicated in the disruption of neuroblastoma ras viral oncogene homolog (NRAS), a key member of the RAS family well-known for its GDP/GTP-regulated on/off switch mechanism. This disruption subsequently activates the mitogen-activated protein kinase/extracellular signal-regulated kinase (MEK1/ERK1–2) oncogenic signaling pathway and contributes to glioma progression and resistance to TMZ therapy (46). Research revealed that circKIF4A was remarkably elevated in glioma tissues and cell lines (47), which resulted in glioma progression and TMZ resistance. Mechanistically, circKIF4A, by interacting with miR-335-5p, regulates the expression of a glycolysis-regulating enzyme aldolase A (ALDOA). The outcomes would be an elevated glycolysis rate, enhanced glioma growth, and increased resistance to TMZ (47). Furthermore, suppression of circKIF4A significantly increases the TMZ- sensitivity both *in vitro* and *in vivo* (47). A study showed that Circ-VPS18 expression was remarkably elevated in TMZ-resistant and glioma tissues (48). The interaction of Circ-VPS18 with miR-370 downregulates runt-related transcription factor 1 (RUNX1) expression, a member of the RUNX transcription factors with oncogenic properties, and subsequently accelerates the glioma growth as well as TMZ-resistance (48). Furthermore, the suppression of Circ-VPS18 significantly increases glioma responsiveness to the TMZ *in vivo* (48). Deng *et al*. have shown the upregulation of circ_0005198 expression in TMZ-resistant glioma cells, glioma tissues, and serum (46). Suppression of circ_0005198 reverses TMZ resistance and increases the apoptosis of TMZ-resistant glioma cells (46). This effect was mediated through circ_0005198 targeting of MiR-198 and further regulation of MiR-198 target gene tripartite motif-containing 14 (TRIM14), which is involved in EMT and chemoresistance of glioma (46, 49). Dysregulation of the RTK/PI3K signaling pathway is deemed to be one of the principal pathways mediating glioma therapeutic resistance. Using high-throughput RNA sequencing, circHIPK3 expression has been shown to be increased in TMZ-resistant glioma cells that act as hsa- miR-524-5p sponge. This facilitates glioma progression and resistance through up-regulating an M-type nonmotile microtubule depolymerase, kinesin family member 2A (KIF2A), expression through activating the PI3K/AKT pathway (50). Furthermore, hsa_circ_0110757 exhibits upregulation in TMZ-resistant glioma cells, operating as a sponge for hsa-miR-1298-5p. This activity promotes glioma progression and resistance by augmenting integrin subunit alpha 1 (ITGA1) gene expression by activating the PI3K/AKT pathway (51). Since the PI3K/AKT pathway has a close relationship with apoptosis-related proteins, it could be concluded that hsa_circ_0110757 induced glioma resistance to TMZ is partially mediated through the regulation of apoptosis. CircRNAs also work as miRNA sponges to impact glioma apoptosis and subsequent TMZ sensitivity by regulating sirtuin 1 (SIRT1) expression. SIRT1 is a NAD-dependent deacetylase with oncogenic and epigenetic regulatory functions. SIRT1 also induces pro-survival and anti-apoptosis properties in different cancer types (52). Circ_0076248 acts as a miR-181a sponge to assist TMZ chemotherapy resistance by increasing the expression of SIRT1 (53). Circ_CEP128 is up-regulated in TMZ-resistant glioma cells. On the other hand, the knockdown of circ_CEP128 inhibits cell proliferation and increases TMZ sensitivity by interacting with miR-145-5p, further decreasing the expression of ATP-binding cassette superfamily G member 2 (ABCG2) (54). Another circRNA, circ-GLIS3, a miR-548m sponge, is also up-regulated in TMZ-resistant glioma cells and facilitates glioma progression and resistance presumably through the induction of mediator of RNA polymerase II transcription subunit 31 (MED31) expression (a transcription coregulatory) (55). Taken together, all this evidence revealed the positive regulatory effects of circRNAs on glioma TMZ resistance and offered circRNAs as ideal biomarkers for glioma resistance, diagnosis, and treatment.

***Exosomal circRNAs and chemoresistance***

Exosomes, which are nanosized extracellular vesicles carrying diverse cargo, including lncRNAs, miRNAs, and circRNAs, can impact the development as well as the treatment of various malignancies, including gliomas. circRNAs can be transferred by exosomes and contribute to drug resistance by sponging miRNAs. In this line, a study revealed that circ_0072083 is elevated in TMZ-resistant glioma tissues and cells (56). Exosomal circ_0072083 controls Nanog Homeobox (NANOG) expression, a key stemness marker involved in TMZ resistance, via controlling miR-1252-5p-mediated degradation and methylation after targeting N6-methyladenosine (m6A) demethylase, human AlkB homolog (5ALKBH5) (57). ALKBH5 maintains the glioma tumorigenicity and TMZ resistance by regulating resistance-related mRNA (58, 59). Moreover, they found that releasing exosomal hsa_circ_0072083 in resistant cells relies on the Warburg effect. Geng *et al*. demonstrated that exosomal circWDR62 acts as a miR-370-3p sponger to control O-6-Methylguanine-DNA Methyltransferase (MGMT), which is a DNA repair enzyme implicated in chemoresistance and ultimately enhances resistance to TMZ and promotes glioma progression *in vitro* and *in vivo* (30).

Li and colleagues also explored the TMZ resistance regulatory effect of exosomal circ_0043949 (60). They observed that circ_0043949 is extremely expressed in exosomes extracted from TMZ-resistant cells, and exosomal circ_0043949 promotes the resistance of GBM cells to TMZ *in vivo* (60). Functionally, current circular RNA promoted TMZ resistance by up-regulating the expression of integrin alpha 1 (ITGA1) through the sequestration of miR-876-3p. This evidence suggests that ITGA1 is a possible target for overcoming TMZ resistance in GBM (60). It has been indicated that exosomal circ-HIPK3 acts with miR-421 to induce resistance to TMZ by elevating the expression of a well-known tumorigenic transcription factor, the zinc finger of the cerebellum 5(ZIC5) (61, 62). It has been proposed that heparinase is required for the secretion and function of exosomes. Notably, heparanase is positively associated with TMZ resistance, which might be attributed to increased delivery of exosomal circRNAs by heparanase (63). Accordingly, research showed that heparanase facilitates exosomal hsa_circ_0042003 transport from TMZ-resistant glioma cells to drug-sensitive tumor cells, confirming its contribution to TMZ resistance (63). Circ-Serpine2, a GSC exosome-containing circRNA, enhanced glioma progression through the miR-124-3p/KIF20A axis(64). All these exosomal circRNA could be considered a promising biomarker for evaluating TMZ efficacy.

***circRNAs and autophagy-related chemoresistance***

Regulation of autophagy by circRNAs has been proposed as another mechanism involved in regulating chemoresistance (65, 66). Autophagy is an intracellular self-degradation process known to have a dual role in cellular functions and homeostasis by eliciting both cytoprotective and pro-apoptotic effects depending on the cellular context (67, 68). Since appropriate autophagic activity induces cytoprotective functions and makes tumor cells resistant to apoptosis, excessive autophagy results in more apoptosis in tumor cells and consequently reinforces tumor responsiveness to chemotherapy (69). Recently, the role of several autophagy targeting circ-RNAs involved in glioma progression and resistance has been investigated. In this line, Chi *et al*. explored the biological effects of Matrine, a traditional Chinese medicine, on the glioma cells (70). They found that Matrine triggers apoptosis and autophagy due to reduced expression of circ-104075 through suppression of Wnt-β-catenin and PI3K/AKT pathways in glioma cells. circRNA-104075 overexpression counteracts the promoting effects of Matrine on apoptosis and autophagy (70). CircMMP1 is up-regulated in glioma cells and tissues, and the circMMP1 knockdown reduced glioma progression and increased apoptosis and autophagy by targeting the miR-195-5p/TGIF2 axis (70). These results support the pro-apoptotic effects of autophagy and the negative regulation of autophagy by circRNAs. However, circRNAs may positively regulate autophagy in glioblastoma cells, further evoking pro-apoptotic effects and sensitizing glioma cells to chemo/radiotherapy. In an investigation, Hsa_circ_0072309 promoted autophagy and improved the sensitivity of GBM to TMZ in wild type p53 group, but not in the GBM group possessed a p53 mutation via affecting the p53 signaling pathway. They showed that Hsa_circ_0072309 inhibits p53 ubiquitination using MiR-100 and strengthens the durability of p53 protein in the wild-type p53 group. Autophagy suppressant or P53 suppressant could reduce the effect of hsa_circ_0072309 on TMZ sensitivity in p53 wild-type GBM (71). CircRNAs can also affect appropriate autophagy and thus regulate drug resistance. Zhang *et al*. indicated that Hsa_circ_0075323 is considerably expressed in GBM cells and is involved in GBM progression through the regulation of Protein p62 (sequestosome 1) -mediated autophagy pathway (72). p62 plays a vital part in protective autophagy in tumor cells. The author concluded that since protective autophagy is common in chemo/radiotherapy resistance of GBM cells, hsa_circ_0075323 can be considered as a prognostic biomarker or a promising therapeutic target to combat chemo/radiotherapy resistance (72). These studies imply a binary interplay between circ-RNAs and autophagy affecting glioma progression and therapeutic resistance positively or negatively. 

**CircRNAs involved in glioma radioresistance**

Radioresistance is a serious hindrance to clinical glioma therapies due to improved DNA repair, modified DNA damage response (DDR), and evasion of apoptosis (73). Differential expression of circRNAs between radioresistant and radiosensitive patients have been identified. Therefore, the discovery of circRNAs involved in radioresistance or radiosensitivity is essential to improve the outcome of radiotherapy in glioma patients. Several radioresistance-promoting circRNAs have been identified in glioma. According to a study, circATP8B4 was significantly up-regulated in radiation-resistant glioma cells (74). Further evaluations showed that circATP8B4 may perform as a molecular sponge for miR766 to expedite cell radioresistance (74). Ring finger protein 2 (RNF2) is an eminent E3 ligase with oncogenic proprieties and is closely linked to the ubiquitination and phosphorylation of the DNA damage molecule, H2AX. H2AX is a pivotal sensor that can provoke the early DDR. RNF2 elicits tumor-promoting functions following exposure to irradiation (75). Accordingly, silencing RNF2 sensitizes glioma cells to radiation by enhancing apoptosis (76). Hence, identifying RNF2-associated circRNAs is important for overcoming glioma radioresistance. Di and colleagues have consistently found that circ_0008344 level is highly elevated in radioresistant glioma cells and tissues. On the other hand, suppression of circ_0008344 enhanced glioma radiosensitivity both *in vitro* and *in vivo* (77). Functionally, circ_0008344 performed as a miR-433-3p sponge to improve glioma radioresistance by augmenting the Ring finger protein 2 (RNF2) expression (77-79). Evidence suggests that radiation can cause the release of exosomes containing oncogenic circRNAs, which can promote proliferative and resistant profiles, in conformity with this notion. Wang *et al*. indicated that low-dose irradiation can cause the release of exosomes containing circ-METRN. Up-regulated circ-METRN increases the gamma H2A histone family member X ( γ-H2AX) expression and stimulates the DDR process in radioresistant glioblastoma cells (80). A study revealed that CircATP8B4 in extracellular vesicles obtained from radioresistant cells reduces radiation sensitivity via sponging miR-766 (74). In contrast to the above-mentioned radioresistant promoting circRNA, the effects of circ-AKT3 as radio-sensitizing circRNA in glioma cells have been examined by Xia *et al*. (81). They demonstrated that circ-AKT3 is downregulated in GBM and acts as a tumor suppressor. Circ-AKT3 encodes a 174 amino acid (aa) novel protein, AKT3-174aa, which can reduce GBM cell growth, *in vivo* tumorigenicity, and radiation resistance. Regarding the mechanism of action, AKT3-174aa competitively cooperates with phosphorylated PDK1, decreases AKT-thr308 phosphorylation, and thus negatively regulates the RTK/PI3K signaling pathway (81). According to these findings, circ-AKT3 can induce AKT3-174aa, a negative RTK/PI3K signaling regulator. Hence, circRNAs have a dual regulatory effect in glioma radioresistance mechanisms. Together, these findings highlight the promise of circRNAs as a promising diagnostic tool for overcoming radioresistance in glioma radiotherapy.

**CircRNAs involved in glioma targeted therapy- resistance**

Platinum chemotherapeutic drugs, particularly Cisplatin (DDP), are broadly applied for treating multiple tumors, such as gliomas. DDP indicates a cytotoxic effect through the construction of the platinum-DNA complex, resulting in the activation of apoptotic signaling pathways (82, 83). Unfortunately, tumor cells are prone to evolve resistance to DDP treatment, which is a main obstacle to glioma therapy (84). Meanwhile, circRNAs have also been reported to regulate glioma resistance to DDP. In this line, researchers examined the effect of circ_PTN in developing resistance to DDP in GBM cells (85). circ_PTN was up-regulated in DDP-resistant GBM cells and enhances resistance to DDP in these cells via sponging miR-542-3p and up-regulating Phosphatidylinositol 3-kinase regulatory subunit gamma (PIK3R3), thereby activating PI3K/AKT signaling pathway(85). Circ_0055412 is another circRNA reported to be significantly up-regulated in glioma cells (86). Suppression of circ_0055412 enhances cisplatin sensitivity of glioma cells *in vitro* and *in vivo*. This circular RNA affects miR-330-3p to simplify cisplatin resistance in glioma cells by increasing the expression of the regulated nuclear factor of activated T cells 3 (NFATC3)(86). NFATC3 can activate the transcription of catenin beta 1 (CTNNB1) to increase β-catenin and stimulate the Wnt/β-catenin signaling pathway(86).

Histone deacetylases (HDACs), the chief arms of the epigenetic control of gene expression, play a crucial role in glioma progression and resistance to treatment (87, 88). In consequence, suppressing HDACs is a promising therapeutic approach for reversing epigenetic modification in glioma. However, resistance to (HDAC) inhibitor-based therapeutic drugs is one of the foremost difficulties in glioma therapy. CircRNAs have been postulated to be implicated in the modulation of HDAC inhibitor tolerance in multiple human malignancies and gliomas. In this line, Meng *et al*. explored the association between circRNAs and tolerance to a pan-HDAC inhibitor, SAHA (Vorinostat), in GBM (89). They discovered circ_0000741, and TRIM14 were up-regulated, while miR-379-5p was downregulated in SAHA-tolerant GBM cells. Furthermore, circ_0000741 depletion inhibited cell proliferation and invasion, reduced SAHA tolerance, and promoted apoptosis in SAHA-tolerant GBM cells (89). Circ_0000741 operated as a miR-379-5p sponge, influencing TRIM14. Thus, Circ_0000741 might promote SAHA tolerance via modulation of the miR-379-5p/TRIM14 axis, offering a promising target for treating GBM (89). Therefore, circRNA targeting might provide a new approach for glioma-targeted therapy. A summary of circRNAs and potential underlying mechanisms in the development of glioma therapeutic resistance is listed in Table 1. 

**circRNAs involved in glioma therapeutic resistance through regulation of GSCs**

It has been evidenced that GSCs, a minor population of cells with stem cell-like properties, have self-renewal and multilineage differentiation potential, contributing to GBM therapeutic resistance and recurrence (90). GSCs promote therapeutic resistance by induction of the DNA damage machinery response and activation of several chemo-resistance-mediating factors and signaling pathways (90-94). Several dysregulated signaling pathways, such as Notch, NF-κB, PI3K/Akt/mTOR, and Wnt/β-catenin, are accountable for tumorigenesis and therapeutic resistance of GBM by GSCs (95-101). circRNAs have been shown to contribute efficiently to the modulation of GSCs maintenance and carcinogenesis, thereby affecting strategies for anti-glioma therapy. Given that GSC features are closely associated with glioma tumorigenesis and therapeutic resistance, identifying GSCs-associated circRNAs and the mechanism of their action will be of great clinical significance in improving anti-glioma treatments. Accordingly, in the following sections, circRNA-mediated regulation of stemness properties, regulation of deregulated transcription factors, and signaling pathway in GSCs and their functional relevance in GSCs maintenance and carcinogenesis will be discussed. 

***Regulation of the expression of stemness markers***

Several findings show the role of circRNAs in regulating stemness properties and subsequent GSC maintenance and carcinogenesis. In this context, a study revealed that circPTN sponges miR-145-5p/miR-330-5p to increase the self-renewal of GSCs and the expression of different stemness markers (Nestin, CD133, SOX9, and SOX2) (102). circEPHB4 has been also shown to sponges miR-637 and further increase the expression of stemness markers, SOX10 and Nestin(103). This effect is associated with enhanced glioma cell stemness, proliferation, and glycolysis(103). circNDC80 has been found as an oncogenic factor in the development of glioblastoma through the miR-139-5p/ Endothelin-converting enzyme-1 (ECE1) pathway that preserves the stemness of GSCs to enhance GSC self-renewal (104). ATP-binding cassette, subfamily C, member 3 (ABCC3), is a member of the ABC transporter superfamily that is involved in multidrug resistance. circABCC3 sponges miR-770-5p to increases the expression of SOX2 stemness marker through the PI3K/AKT pathway in glioma cells(105). The SOX family transcription factors have been found to be fundamental regulators of stemness, EMT, carcinogenesis, and drug resistance. Thus, circRNA-mediated targeting of SOX transcription factors may be a useful target for overcoming GSC maintenance and resistance.

***Regulation of the expression of other GSC markers***

Along with their role in regulating stemness properties described above, circRNAs also regulate resistance-mediating factors in GSCs. Based on a report, circCHAF1A expression was elevated in glioma and enhanced the proliferation and tumorigenesis of GSCs(106). Regarding its mechanism of action, circCHAF1A increased the expression of transcription factor Homeobox C8 (HOXC8) in GSCs by targeting miR-211-5p. Up-regulated HOXC8 can, in return, increase the MDM2 expression, which is highly associated with chemotherapeutic resistance in human malignancies, and suppress the antitumor effect of p53. Additionally, the RNA-binding protein FMR1 can bind to and enhance the expression of circCHAF1A by preserving its stability, while HOXC8 also transcribes the FMR1 expression to create a feedback loop which ultimately may facilitate the tumorigenesis of GSCs(106). circASPM has been shown to be overexpressed in GBM, enhancing GSC proliferation and tumorigenesis (107). Functionally, circASPM directly binds to miR-130b-3p as a molecular sponge in GSCs. This binding induces overexpression of E2 transcription factor1 (E2F1), a regulator of tumor occurrence and chemoresistance, ultimately enhancing GSC proliferation and tumorigenesis(107, 108). Another circRNA, circ-ASB3, has been presented to be up-regulated in GSCs, increasing the expression of Twist1, a master regulator of EMT, by competitively inhibiting miR-543, thereby enhancing glioma malignancy and recurrence(109). circPTPRF enhances the progression and neurosphere formation ability of GBM by sponging miR-1208 to augment the expression of the Yin Yang 1 (YY1) transcription factor (110). Another investigation revealed that circMELK is up-regulated in GBM, acting as a sponge for tumor suppressor miR-593, to increase the oncogenic gene Eph receptor B2 (EphB2), a member of Eph receptor tyrosine kinases family identified as a regulator of cancer stemness, drug resistance and subsequently glioblastoma EMT and GSC maintenance (105). Similarly, circRNAs, such as circARF1, circATP5B, and circCHAF1A, are overexpressed in GBM and involved in GSC proliferation and tumorigenesis (106, 111). circARF1 through U2AF2 /circARF1/miR-342-3p/ ISL LIM homeobox 2 (ISL2) feedback loop affects angiogenesis in GSCs(111). Experiments proved that circARF1 increased the expression of ISL2 in GSCs via miR-342-3p sponging. Additionally, U2AF2 binds to and enhances the stability and expression of circARF1, while ISL2 stimulates U2AF2 expression, which creates a feedback loop in GSCs(111). From these documents, it could be concluded that circRNAs, by sponging miRNAs and subsequent alteration of gene expression, create a feed-forward regulatory loop to increase their stability and expression in GSCs.

***Regulation of dysregulated signaling pathways in GSCs***

CircRNA-mediated targeting of dysregulated signaling pathways may represent another GSC maintenance and resistance mechanism. According to Hu *et al*., circGNB1 is up-regulated in glioma, promoting GSC proliferation, invasion, and neurosphere formation (112). Functional assays have claimed that circGNB1 acts as miR-515-5p and miR-582-3p sponge to increase the expression of oncogene Xenotropic and polytropic retrovirus receptor 1 (XPR1), an inorganic phosphate cell-surface transmembrane protein inducing chemotherapy resistance by reducing Pi concentration (112, 113). XPR1 could further promote the malignant phenotype of GSCs through IL6 overexpression and JAK2/STAT3 signaling activation (112). Consistently, circATP5B has been reported to be up-regulated in GSCs, promoting the proliferation of GSCs through activation of the IL6-mediated JAK2/STAT3 signaling pathway (114). circRPPH1 had also proved to be up-regulated in GSCs, promoting the self-renewal and proliferation of GSCs by up-frameshift protein 1 (UPF1)/circRPPH1/ Activating transcription factor (ATF3) feedback loop through interacting with the TGF-β signal pathway (115). Furthermore, the knockdown of circRPPH1 markedly reduced the GSC proliferation and clonogenicity(115). In a recent work, circNCAPG has been found to be overexpressed in glioma, contributing to GSC progression(64). Functionally, circNCAPG interacts with and stabilizes a zinc finger transcription factor, RAS-responsive element-binding protein 1 (RREB1), that can activate the TGF-β1 signaling pathway. Furthermore, RREB1 could surge the GSC stemness through overexpression of Nestin and also elevate the expression of the U2AF65 splicing factor to develop the stability of circNCAPG in GSCs(64). Jiang *et al*. demonstrated that over-expressed circKPNB1 in GBM can promote the GSC tumorigenicity, neurosphere formation abilities, and stemness. They found that circKPNB1 controls the protein stability and nuclear translocation of transcription factor SPI1 so that SPI1 enhances the malignant phenotype of GSCs through TNF-α mediated NF-κB signaling(116). Evidence suggests that circRNA-encoded proteins could also contribute to glioma tumorigenesis and development. For instance, circRNA, circSmo encodes a novel protein Smoothened (SMO) -193a.a, essential for Hedgehog signaling activation in CSCs (117). Up-regulation of Smo-193a.a is linked to abysmal prognosis of glioblastoma and positively controls the Hedgehog signaling, while the downregulation of Smo-193a.a can considerably suppress self-renewal of CSCs and tumorigenesis (117). Irregular activation of Growth Factor Receptor (EGFR) signaling is common in GBM and is associated with up to 60% of all GBM cases(118, 119). EGFR is also involved in GSCs maintenance and functions, and acquired resistance to EGFR Inhibitors appears to be associated with GSCs. According to Gao *et al*., the circ-E-Cad variant activates EGFR independently of EGF and GSC tumorigenicity maintenance (120). They also found that inhibition of circ-E-Cad meaningfully enhances the anticancer effects of EGFR-targeting therapies in GBM (120). These findings underscore the therapeutic potential of circRNAs in EGFR-driven GBM. Together, these collective findings suggest that GSCs-associated circRNAs can regulate stemness properties by targeting the specific gene and the downstream signal pathways known to mediate glioma carcinogenesis and resistance. Thus, the merits of targeting GSCs-associated circRNAs should be considered in the context of approaches that target therapy resistance in glioma. Several circRNA--miRNA-target gene interactions affect different characteristics of GSCs in glioma (Table 2**)**.

***Targeting ferroptosis in GSCs***

Ferroptosis is a type of ferrous iron-dependent regulated cell death pathway and assumes a significant character in GBM malignancy. It must be noted that agents inducing the ferroptosis pathway have also been established to increase GBM drug sensitivity (121-123). Accordingly, several circRNA targeting ferroptosis have been recently uncovered. In this line, circCDK14 has been revealed to suppress ferroptosis and increase GBM progression through the regulation of PDGFRA (124). Circ-TTBK2 also modulates glioma cell tumorigenicity and ferroptosis via targeting ITGB8 by sponging miR-761 (125). Knockdown of specific circRNA increases ferroptosis and enhances drug sensitivity in other types of tumors (126). An important note, due to certain features of CSCs, ferroptosis can selectively target aggressive CSCs and induce CSCs death in tumors (127, 128). Therefore, identifying ferroptosis-targeting circRNAs in GSCs could potentially be exploited to eradicate GSCs and suppress glioma progression and resistance. In conformity with this notion, Jiang *et al*. indicated diminished expression of circLRFN5 in GBM, and that circLRFN5 overexpression reduces the GSC proliferation, stemness, and tumorigenesis through induction of ferroptosis(129). They reported that CircLRFN5 connects to paired related Homeobox 2(PRRX2) protein, promoting its proteasomal degradation and suppressing PRRX2-mediated transcription of Guanosine triphosphate cyclohydrolase (GCH1), a ferroptosis suppressor, in GSCs(129). Thus, circRNA-mediated ferroptosis regulation may serve as a novel GSCs-targeting therapy and, thus, overcome therapeutic resistance.

**Future perspectives**

**Figure 2:**
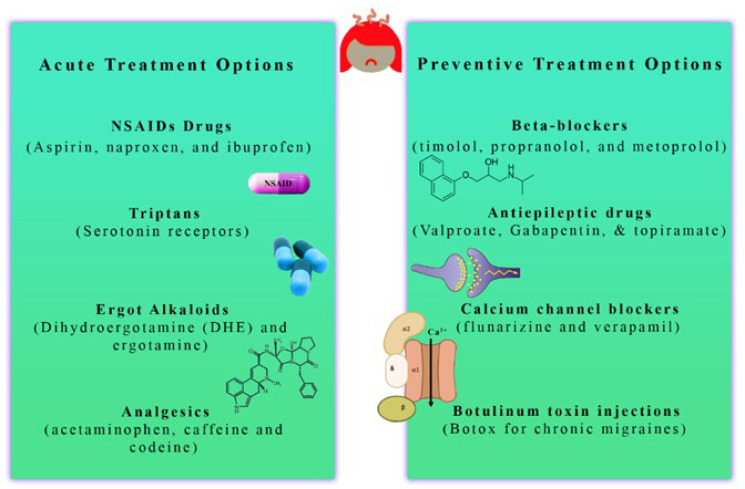
A diagrammatic illustration of resistance regulatory mechanisms of circRNAs in glioma by affecting cell apoptosis, autophagy, EMT, ferroptosis, exosomes, and stemness

As we have summarized current knowledge on the regulatory effects of circRNAs in therapy resistant-glioma, it seems that circRNAs perform as a double-edged sword in therapeutic resistance. Most of the circRNAs mentioned in this review are overexpressed in glioma and contribute to therapeutic resistance by activation of divers signaling pathways, including the PI3K/AKT, Wnt-β-catenin, Notch and NF-κB, thus affecting cell survival, apoptosis, autophagy, EMT, and stemness. A graphical presentation is provided in Figure 2. For these circRNAs that promote therapeutic resistance, targeted inhibition or an appropriate combination of circRNA-targeted agents and chemo/radiotherapy may present a viable strategy for increasing tumor sensitivity. Several RNA-based therapies, such as antisense oligonucleotide and interfering RNA, have gained FDA approval (131). However, the therapeutic targeting of circRNAs is still at an early stage and is far from being able to be incorporated into clinical settings and requires further investigations. Some circRNAs are downregulated in glioma and play a tumor-suppressive role in glioma by inhibiting therapeutic resistance. Endogenous or synthetic circRNAs, therefore, have the potential to be used as a potent therapeutic sensitizer. The successful application of circRNA overexpression strategies is also limited and requires complementary research. For example, the appropriate doses and the immunogenic and toxic effects of circRNAs have to be determined and optimized in target cells. Identifying a standardized circRNA delivery system can reduce the limitations of circRNA-based approaches caused by off-target effects. Exosomes represent a novel means of intercellular communication by delivering various circRNAs that affect post-transcriptional genetic regulation to exert glioma growth and resistance. Thus, following up with further preclinical validation and clinical exploration of exosomal circRNAs in human gliomas would be worthwhile in developing more effective diagnostic and therapeutic strategies. Abnormal expression of circRNAs can potentially be a diagnostic biomarker of therapeutic resistance. Applying circRNAs as potential biomarkers would help choose patients most likely to benefit from chemotherapy and radiation therapy. Despite the discovery of many circRNAs, there are still many circRNAs with unknown functions in glioma therapeutic resistance, especially GSCs. It is important to note that autophagy is related to processes that drive GSC maintenance and tumorigenicity. Thus, elucidating the cross-talk between circ-RNAs and autophagy in GSCs could be beneficial for establishing more qualified therapeutic modalities for controlling chemo/radioresistance. Mechanisms underlying the effect of the circRNAs on therapeutic resistance in tumors are complex. Further identification of the effects of circRNAs on other resistance-promoting factors and signaling pathways is therefore worthy of consideration. 

CircRNAs play key roles in glioma cellular proliferation, angiogenesis, and invasion by regulating the complementary miRNAs or target mRNAs

**Table 1:** CircRNAs and their mechanisms in the development of glioma therapeutic resistance

CircRNA	miRNA	Target gene	Expression	Biological Effect	Treatment	Study design	Ref.
hsa_circ_0000936	miR-1294		Up	Chemoresistance	TMZ	*in vitro*	(45)
circASAP1	miR-502-5p	NRAS	Up	Induction of cell growth, inhibition of apoptosis activation of MEK1/ERK1–2	TMZ	*in vitro* /*in vivo*	(46)
circKIF4A	miR-335-5p	ALDOA	Up	Increasing the glycolysis rate, glioma growth, and chemoresistance	TMZ	*in vitro* /*in vivo*	(47)
circ-VPS18	miR-370	RUNX1	Up	Increasing the glioma growth and chemoresistance	TMZ	*in vitro* /*in vivo*	(48)
circ_0005198	miR-198	TRIM14	Up	Increasing the EMT and chemoresistance	TMZ	*in vitro*	(46)
circHIPK3	hsa- miR-524-5p	KIF2A	Up	Increasing the glioma progression and resistance Activating the PI3K/AKT	TMZ	*in vitro*	(50)
hsa_circ_0110757	hsa-miR-1298-5p	ITGA1	Up	Regulating apoptosis Activating the PI3K/AKT pathway	TMZ	*in vitro* /*in vivo*	(51)
circ_0076248	miR‐181a	SIRT1	Up	Increasing the glioma growth, invasion, and resistance	TMZ	*in vitro* /*in vivo*	(53)
circ_ CEP128	miR-145-5p	ABCG2	Up	Increasing the glioma cell proliferation and resistance	TMZ	*in vitro* /*in vivo*	(54)
circ-GLIS3	miR-548m	MED31	Up	Increasing the glioma progression and resistance	TMZ	*in vitro* /*in vivo*	(55)
Exosomal circ_0072083	miR-1252-5p	(NANOG)	Up	Increasing the glioma tumorigenicity	TMZ	*in vitro* /*in vivo*	(56)
Exosomal circWDR62	miR-370-3p	MGMT	Up	Increasing the glioma tumorigenicity and resistance	TMZ	*in vitro* /*in vivo*	(30)
Exosomal circ_0043949	miR-876-3p	ITGA1	Up	Increasing the glioma progression and resistance	TMZ	*in vivo*	(60)
Exosomal circ-HIPK3	miR-421	ZIC5	Up	Increasing the glioma tumorigenicity and resistance	TMZ	*in vitro* /*in vivo*	(61)
circ-Serpine2	miR-124-3p	KIF20A	Up	Increasing the glioma cell proliferation, migration, invasion, and resistance	TMZ	*in vitro* /*in vivo*	(64)
circ- ATP8B4	miR 766		Up	Increasing the glioma resistance	Radiotherapy	*in vitro* /*in vivo*	(74)
circ_0008344	miR-433-3p	RNF2	Up	Increasing the glioma tumorigenicity and resistance	Radiotherapy	*in vitro* /*in vivo*	(77)
circ-METRN		γ-H2AX	Up	Promoting the DDR process and resistance	Radiotherapy	*in vitro*	(80)
circ-AKT3		protein, AKT3-174aa	Down	Reducing GBM cell proliferation and resistance	Radiotherapy	*in vitro* /*in vivo*	(81)
hsa_circ_0003949	miR-542-3p	PIK3R3	Up	Increasing the glioma tumorigenicity and resistance Activating the PI3K/AKT pathway	Cisplatin	*in vitro* /*in vivo*	(85)
circ_0055412	miR-330-3p	NFATC3	Up	Increasing the glioma resistance Activating the Wnt/β-catenin signaling pathway	Cisplatin	*in vitro* /*in vivo*	(86)
circ_0000741	miR-379-5p	TRIM14	Up	Increasing the glioma cell proliferation and invasion and reducing apoptosis	Vorinostat	*in vitro* /*in vivo*	(89)
circ-104075			Up	Reducing apoptosis and autophagy, Activating the Wnt /β-catenin and PI3K/AKT signaling pathways	Matrine	*in vitro*	(70)
hsa_circ_0072309	miR-100		Down	Promoting autophagy and TMZ sensitivity	TMZ	*in vitro* /*in vivo*	(71)
hsa_circ_0075323		Protein p62	Up	Promoting autophagy Increasing the GBM progression and resistance	TMZ	*in vitro*	(72)

Temozolomide (TMZ), neuroblastoma ras viral oncogene homolog (NRAS), aldolase A (ALDOA), runt-related transcription factor 1 (RUNX1), tripartite motif-containing 14 (TRIM14), kinesin family member 2A (KIF2A), sirtuin 1 (SIRT1), integrin subunit alpha 1 (ITGA1), ATP-binding cassette superfamily G member 2 (ABCG2), mediator of RNA polymerase II transcription subunit 31 (MED31), Nanog Homeobox (NANOG), O6-methylguanine-DNA methyltransferase (MGMT), zinc finger of the cerebellum 5(ZIC5), Ring finger protein 2 (RNF2), the gamma H2A histone family member X ( γ-H2AX), Phosphatidylinositol 3-kinase regulatory subunit gamma (PIK3R3), regulated nuclear factor of activated T cells 3 (NFATC3)

**Table 2:** Summary of circRNAs--miRNA-target gene interactions affecting GSCs in glioma

circRNA	microRNA	Target gene	Expression	Biological Effects	Pathway	Ref.
circATP5B	miR-185-5p	HOXB5	Up	GSC self-renewal and proliferation	IL6-mediated JAK2/STAT3	(114)
circRPPH1	-	UPF1 ATF3	Up	GSC self-renewal, proliferation, clonogenicity	TGF-β signaling pathway	(115)
circNCAPG	-	RREB1	Up	GSCs progression and GSC stemness	TGF-β1 signaling pathway	(64)
circKPNB1	-	SPI1	Up	GSC stemness tumorigenicity	TNF-α mediated NF-κB signaling	(116)
circSmo		SMO-193a.a	Up	Self-renewal of CSCs and tumorigenesis	Hedgehog signaling	(117)
circ-E-Cad	-	Protein C-E-Cad	Up	GSC tumorigenicity	EGFR signaling	(120)
circPTN	miR-145-p, miR-330-5p	Nestin, CD133, SOX9, SOX2	Up	GSC self-renewal		(102)
circEPHB4	miR-637	SOX10, Nestin	Up	Glioma cell stemness, proliferation, and glycolysis		(103)
circNDC80	miR-139-5p	Endothelin converting enzyme 1	Up	GSCs stemness and self-renewal, cell proliferation, migration, invasion	ECE1 pathway	(104)
circCHAF1A	miR-211-5p	HOXC8	Up	GSCs proliferation and tumorigenesis		(106)
circASPM	miR-130b-3p	E2F1	Up	GSC proliferation, tumorigenesis		(107)
circ-ASB3	miR-543	Twist1	Up	Glioma malignancy and recurrence		(109)
circPTPRF	miR-1208	YY1	Up	Glioma neurosphere formation ability and progression		(110)
circMELK	miR-593	EphB2	Up	EMT and GSC maintenance		(105)
circARF1	miR-342-3p	ISL2	Up	Angiogenesis in GSCs		(111)
circGNB1	miR-515-5p, miR-582-3p	XPR1	Up	GSC proliferation, invasion, and neurosphere formation	IL6-mediated JAK2/STAT3	(112)
circLRFN5	-	PRRX2	Down	GSCs proliferation, stemness, tumorigenesis	Ferroptosis pathway	(130)

glioma stem cells (GSCs), Endothelin-converting enzyme-1 (ECE1), homeobox B5 (Hoxb5), up-frameshift protein 1 (UPF1, Activating transcription factor (ATF3), RAS-responsive element-binding protein 1 (RREB1), Protein. Spi-1 proto-oncogene (SPI1), Homeobox C8 (HOXC8),E2 transcription factor1 (E2F1),Yin Yang 1 (YY1),Eph receptor B2 (EphB2),ISL LIM homeobox 2 (ISL2), Xenotropic and polytropic retrovirus receptor 1 (XPR1), paired related Homeobox 2(PRRX2)

## Conclusion

Taken together,circRNAs exhibit abnormal expression patterns in glioma and may function as either facilitators or suppressors of therapeutic resistance. circRNAs serve as competing endogenous RNAs, sequestering specific microRNAs and thereby influencing the expression of genes associated with glioma tumorigenesis and resistance. By doing so, circRNAs can alter essential cellular pathways and processes that govern glioma resistance, including DNA repair mechanisms, glioma stem cells, epithelial-mesenchymal transition, apoptosis, and autophagy. Given the dismal survival rates and increased resistance to existing glioma treatments, pursuing further preclinical validation and clinical investigation of circRNAs in human gliomas is imperative to establish more effective diagnostic and therapeutic approaches.

## Data Availability

No datasets were produced in this study.

## Declaration

The authors declare that they have not used any AI tools or technologies to prepare the manuscript.
